# Robot-based assessment of motor and proprioceptive function identifies biomarkers for prediction of functional independence measures

**DOI:** 10.1186/s12984-015-0104-7

**Published:** 2015-11-26

**Authors:** Sayyed Mostafa Mostafavi, Parvin Mousavi, Sean P. Dukelow, Stephen H. Scott

**Affiliations:** School of Computing, Queen’s University, Kingston, ON Canada; Hotchkiss Brain Institute, University of Calgary, Calgary, AB Canada; Department of Biomedical and Molecular Sciences, Queen’s University, Kingston, ON Canada; Providence Care, St. Mary’s of the Lake Hospital, Kingston, ON Canada; Centre for Neuroscience Studies, Queen’s University, Kingston, K7L 3N6 ON Canada

**Keywords:** Stroke assessment, Prognosis, Proprioception, Robotic assessment, Upper limb, Stroke

## Abstract

**Background:**

Neurological impairments following stroke impact the ability of individuals to perform daily activities, although the relative impact of individual impairments is not always clear. Recovery of sensorimotor function following stroke can vary widely, from complete recovery to modest or minimal improvements, across individuals. An important question is whether one can predict the amount of recovery based on initial examination of the individual. Robotic technologies are now being used to quantify a range of behavioral capabilities of individuals post-stroke, providing a rich set of biomarkers of sensory and motor dysfunction. The objective of the present study is to use mathematical models to identify which biomarkers best predict the ability of subjects with stroke to perform daily activities before and after rehabilitation.

**Methods:**

The Functional Independence Measure (FIM) was quantified approximately 2 weeks and three months post-stroke in 61 ischemic and 24 hemorrhagic subjects with stroke. At 2 weeks post-stroke, subjects also completed clinical assessments and robotic assessments of sensory and motor function. A computational search algorithm, known as Fast Orthogonal Search, was used to identify the robotic and clinical biomarkers that best estimated Functional Independence Measures.

**Results:**

Clinical and robot-based biomarkers were statistically similar at predicting FIM scores at 2 weeks (r = 0.817 vs. 0.774, respectively) and 3 months (r = 0.643 vs. 0.685, respectively). Importantly, robot-based biomarkers highlighted that parameters related to proprioception were influential for predicting FIM scores at 2 weeks, whereas biomarkers related to bimanual motor function were influential for predicting FIM scores at 3 months.

**Conclusions:**

The present study provides a proof of principle on the use of robot-based biomarkers of sensory and motor dysfunction to estimate present and future FIM scores. The addition of other behavioral tasks will likely increase the accuracy of these predictions, and potentially help guide rehabilitation strategies to maximize functional recovery.

## Background

A key focus of rehabilitation therapy following stroke is to regain the ability to perform daily activities. The Functional Independence Measure (FIM) is routinely used in Canada and the United States to score rehabilitation inpatients to quantify the ability to perform these daily activities following stroke using a 7-point scale for 13 motor and 5 cognitive tasks such as getting dressed, grooming and bowel control [1]. The FIM was designed to track changes in the functional status of patients prior to and following rehabilitation [2]. FIM scores have been shown to predict functional outcome following rehabilitation [3]. While FIM intake and discharge scores are correlated to some degree [4], predicting future abilities from early FIM scores is problematic - improved FIM scores following rehabilitation can reflect improved motor function in the affected limb (motor recovery), and/or acquired skills to perform motor actions unimanually with the unaffected limb (compensation) [5, 6].

The inability to perform daily activities following stroke is, of course, caused by focal damage in the brain that disrupts circuits that support various sensory, motor and cognitive processes. Several studies highlight that lesion size and location can predict motor recovery [7]. However, such predictions are not straightforward due to the close proximity of key circuits supporting various brain faculties. For example, small focal lesions near the internal capsule and thalamus can lead to varying levels of limb proprioceptive and motor impairments [8].

Another approach to predict future capabilities of daily activities is based on the underlying sensory, motor and cognitive impairments. There is an obvious link between neurological impairments and difficulties in performing daily activities. Measures of motor impairments in the affected arm generally correlate with FIM [4, 9]. As well, proprioceptive impairments also correlate with FIM scores [9, 10]. However, improvements in FIM can occur independent of the affected limb if individuals are able to perform the activity only with their unaffected limb. This unaffected arm often also shows impairments following stroke [11], and thus also potentially impacts functional recovery.

The purpose of this study was to use robot and clinical biomarkers associated with upper limb function quantified in the first few days post-stroke to estimate the ability of subjects to perform daily activities at this same time point, as well as to predict their abilities at 3 months post-stroke. In particular, recent development of robot-based behavioural tasks provide a rich set of biomarkers of sensory and motor function, including performance of the affected and unaffected arms. Our interest was to observe which features of sensory and motor function were most important for predicting both FIM scores prior to and following rehabilitation.

## Methods

### Participants

Subjects with stroke were recruited after admission to St. Mary’s of the Lake Hospital in Kingston, Ontario, Canada, Dr. Vernon Fanning Care Centre (Calgary, AB, Canada), and Foothills Hospital, Calgary, Alberta, Canada for clinical stroke assessment and robotic evaluation. Clinical evaluation sessions were performed within approximately two weeks post-stroke. Initial FIM measurements were also completed approximately two weeks post stroke. Evaluation of the second FIM measurements were completed 3 months+/-1 month post stroke. We henceforth refer to these two FIM evaluations as *FIM-2w* and *FIM-3 m,* respectively. Inclusion criteria were the following: (a) first clinical presentation of stroke; (b) ability to understand the task instructions; and (c) sufficient range of motion to complete robotic testing. Subjects were excluded from the study if they had a history of neurological impairments not caused by stroke, any form of acute medical illness, or musculoskeletal compromise of the shoulder or elbow. Subjects received standard rehabilitation programs that were largely based on principles of motor learning as well as neurodevelopmental techniques. Rehabilitation was generally performed each weekday for 6 to 8 weeks. The study was approved by the ethics review boards of Queen’s University, University of Calgary, and Providence Care, and all subjects for the study gave their informed consent.

### Robotic Assessment

**Fig. 1:**
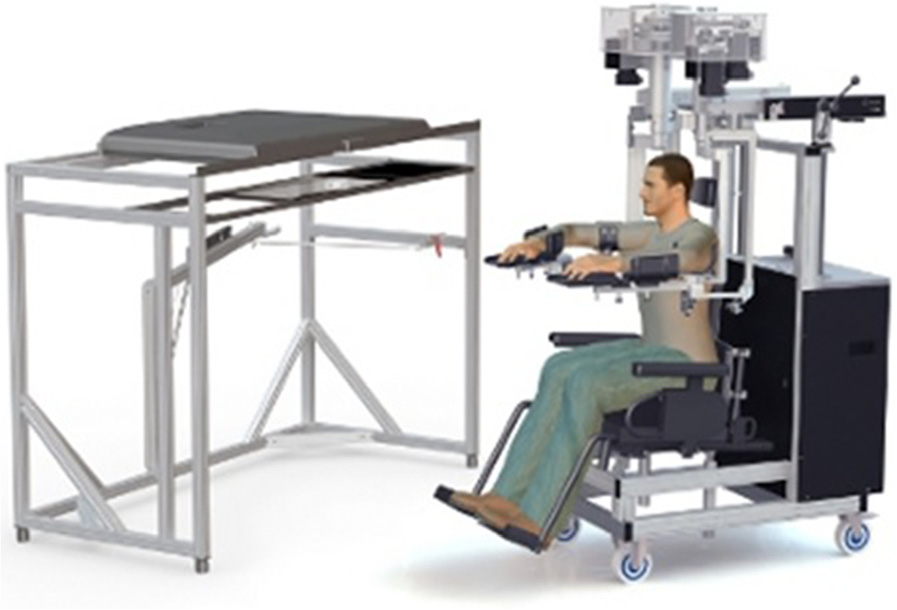
KINARM exoskeleton robotic device used for evaluation of motor and proprioceptive deficits of subjects with stroke

Robotic assessments were performed using the KINARM exoskeleton robotic device (BKIN Technologies, Kingston, Canada, [12]). The subject’s arms were abducted into the horizontal plane. The robot provided gravitational support of the limb, monitored shoulder and elbow motion, and can apply mechanical loads at the shoulder and/or elbow. A virtual reality system aligned with the plane of the arms displayed spatial targets and visual feedback of the hand, as required by each task. Participants did not use the KINARM robot except for the clinical assessments in this study. To avoid fatigue, subjects were allowed to take a break between tasks. Figure 1 depicts a schematic view of the KINARM robot for assessment of a typical subject.

### Visually-Guided Reaching Task

Details of this task and its associated parameters, henceforth referred to as the *Reaching* task, have been described elsewhere [12]. Briefly, subjects were asked to reach “quickly and accurately” from a central target to one of eight peripheral targets located 10 cm away, distributed around the circumference of a circle. There was no emphasis in the task instructions on either speed or accuracy to reach to the targets so each subject selected how to trade-off these task requirements. Each trial began with subjects holding their index finger tip at a central target for 1250-1750 ms. Then a peripheral target was illuminated and subjects were given 3000 ms to complete the reach. Each target was presented once per block and subjects completed eight blocks for a total of 64 trials. Subjects performed this task with both their affected and unaffected arms. A total of 13 parameters were used to quantify subject performance in this task for the affected (RA) and unaffected (RU) arms (See Table 1). For each subject, the value of each measured parameter was averaged across the 64 trials. Interlimb (RI) differences in reaching performance between the two arms were also quantified.

**Table 1:** Abbreviations and description of robotic task parameters for three robotic tasks: visually guided reaching task, arm position matching task and ball drop object hit task

Robotic Task	Parameters	Abbreviation	Brief Description
Visually Guided Reaching	Reaction Time	RA1*	Time between illumination of the peripheral target and onset of movement
	First Movement Max. Speed	RA2	Maximum hand speed during the participant’s initial movement
	First Movement Distance	RA3	Distance the hand traveled during the participant’s initial movement
	First Movement Direction Error	RA4	Angular deviation between (*a*) a straight line from the hand position at movement onset to the peripheral target and (*b*) a vector from the hand position at movement onset to the hand position after the initial phase of movement
	Total Movement Time	RA5	Total time elapsed from movement onset to offset
	Path Length	RA6	Total distance traveled by the hand between movement onset and offset
	Path Length Ratio	RA7	Total distance traveled by the hand between movement onset and offset divided by the straight line distance.
	Movement Time Maximum Speed	RA8	Maximum speed that the hand traveled during the entire reaching movement.
	Number of Movement Peaks	RA9	Number of hand speed maxima between movement onset and offset
	Min. Max. Speed Difference	RA10	Differences between local speed peaks and minima
	First Movement Maximum Speed Ratio	RA11	Ratio of (*a*) the maximum hand speed during the participant’s initial movement to (*b*) the global hand speed maximum of the trial
	First Movement Distance Ratio	RA12	Ratio of (a) the distance the hand traveled during the participant’s initial movement to (b) the distance the hand traveled between movement onset and offset
	Posture Speed	RA13	Mean hand speed for 500 ms before peripheral target illumination
Arm Position Matching	Variability X (m)	M1	Mean of standard deviation of the active hand’s position over all target locations in the *x* direction
	Variability Y (m)	M2	Mean of standard deviation of the active hand’s position over all target locations in the *y* direction
	Variability XY (m)	M3	Mean of standard deviation of the active hand’s position over all target locations in the *xy* direction
	Contraction/expansion ratio X	M4	Range/area of the workspace matched by the active hand relative to that of the passive hand in the *x* direction.
	Contraction/expansion ratio Y	M5	Range/area of the workspace matched by the active hand relative to that of the passive hand in the *y* direction.
	Contraction/expansion ratio XY	M6	Range/area of the workspace matched by the active hand relative to that of the passive hand in the *xy* direction.
	Shift X (m)	M7	Mean of the mean error between the active and passive hands for each target location over all targets in the *x* direction.
	Shift Y (m)	M8	Mean of the mean error between the active and passive hands for each target location over all targets in the *y* direction.
	Shift XY (m)	M9	Mean of the mean error between the active and passive hands for each target location over all targets in the *xy* direction.
Object Hit	Total hits	OH1	Number of balls that are hit and leave the display area of the subject’s workspace
	Hits with Affected Arm	OH2	Number of balls that are hit with the affected arm of stroke participants
	Hits with Unaffected Arm	OH3	Number of balls that are hit with the unaffected arm of stroke participants
	Hand bias of hits	OH4	Normalized difference between the total number of hits with right and left hands
	Miss bias	OH5	Quantifies whether there was a spatial bias in position of balls missed in workspace
	Hand transition	OH6	Line in the workspace where the participant’s preference switches from one hand to the other
	Hand selection overlap	OH7	Quantifies whether both hands share the workspace
	Median error	OH8	Point in the task where the participant missed half of the balls that they missed in the entire task as a percentage of the total number of balls
	Hand speed – Affected Arm	OH9	Average hand speed during the task for the affected arm of subjects with stroke
	Hand speed – Unaffected Arm	OH10	Average hand speed during the task for the unaffected arm of subjects with stroke
	Hand speed bias	OH11	Normalized difference between mean hand speeds of the left and right hands
	Movement area – Affected Arm	OH12	Area of space used by the affected arm of subjects with stroke during the task
	Movement area – Unaffected Arm	OH13	Area of space used by the unaffected arm of subjects with stroke during the task
	Movement area bias	OH14	Quantifies differences in the size of the workspace used by each hand

*RA followed by a number is used for abbreviation of visually guided reaching task parameter on the affected arm. RU and RI followed by a number will be used for the same parameter measured on the unaffected side and the inter-limb difference, respectively

### Arm-Position Matching Task

Details of this task and its associated parameters, henceforth referred to as the *Matching* task, have been described elsewhere [13]. Briefly, subjects allowed the robot to passively move the affected hand (passive arm) to one of nine different spatial locations, arranged as a 3-by-3 square grid, with vision occluded [13]. When the robot stopped, subjects attempted to move the unaffected hand (active arm) to the mirror location in space. Subjects could continue to adjust the position of their active arm until they felt it was mirror-matched with the passive arm position. When subjects indicated they attained the mirror location, they informed the instructor and this was recorded as the final hand position, after which the next trial began. Target locations were such that the outer eight targets were separated by 10 cm. The robot randomly moved from one location to the next. Subjects did not go to a standard start position between trials. Each subject completed six blocks (target locations random within a block) for a total of 54 trials. Nine parameters quantified subject performance (See Table 1).

### Object Hit Task

Details of this task and its associated parameters have been described elsewhere [14]. Briefly, subjects were instructed to use virtual paddles attached to the right and left hands to hit red balls that were moving towards them on the screen. The objective of the task is to hit as many balls as possible. The balls appear on the screen from 10 different (hidden) bins, and a total of 30 balls are released from each bin in random order (all 10 bins release a ball before a bin is reused). Consequently, the game consists of a total of 300 balls falling continuously on the screen. The number of balls that appear on the screen and the speed of the ball movement increases as the task progresses, such that a single ball is moving slowly (~0.01 m/s) at the beginning and up to a maximum of 16 balls moving on the screen at ~0.05 m/s towards the end of the task. The KINARM robot provides force feedback each time a paddle hits a ball. A total of 14 parameters quantified subject performance including total balls hit, as well as spatial and motor features of the task (See Table 1).

### Clinical Measures of Stroke

The Functional Independence Measure (FIM) [15], Purdue Pegboard Test [16], Modified Ashworth Score [17], and Chedoke-McMaster assessment [18] were used for clinical evaluation of subjects with stroke. These measures have established validity and reliability [19, 20].

The FIM measures functional abilities of subjects and is a widely used measure of disability following stroke. Good construct and validity have been established for FIM [21]. The FIM evaluates 18 areas of function, which are rated on a 1–7 scale, ranging from full dependence (1) to full independence with no required assistance (7). The 18 areas of function are categorized into 13 motor items and 5 cognitive items. We refer to the scales corresponding to the 18 items and 13 items as *FIM-Total* and *FIM-Motor*, respectively.

The Purdue Pegboard Test assesses manual dexterity and bimanual coordination [22]. The test involves two different abilities: gross movements of arms, hands, and fingers, and fine motor dexterity. The test consists of taking one peg at a time from a container and placing it into one of the holes on the board as quickly as possible with one hand. The score is expressed in the number of pegs placed in 30 seconds. In the present study, we use the sum of the scores for both hands (referred to as Purdue Combined). Poor Pegboard performance is a sign of deficits in complex, visually guided, or coordinated movements. The test is reported to have good test re-test and inter-rater reliability [23, 24], but suffers from floor effects [21].

The Chedoke-McMaster Stroke Assessment Impairment Inventory is a measure used to assess physical impairment following stroke [18]. In the present study, we added the hand and arm impairment inventory scores, each evaluated on a 7-point scale [25] for both the affected and unaffected limbs (abbreviated to CMS-Affected and CMS-Unaffected, respectively).

Spasticity at the elbow was assessed using the Modified Ashworth Scale [17]. It is an ordinal scale ([0, 1, 1+, 2, 3, 4]), with 0 representing no spasticity. The measurements were performed for the elbow at both the affected and unaffected limbs (abbreviated to MAS-Affected and MAS-Unaffected, respectively).

The conventional subtests of the Behavioral Inattention Test (BIT) are basic pencil and paper measures to screen for visuospatial neglect [26]. A score at or below 129 is indicative of visuospatial neglect.

### Data analysis

We used a normalization scheme to scale all KINARM metrics to values between 0 and 1. The minimum (x_min_) and maximum (x_max_) value of each metric (x) are used to obtain a normalized value, (z(x)), as follows:

1 $$ \mathrm{z}\left(\mathrm{x}\right)=\frac{\mathrm{x}\hbox{-} {\mathrm{x}}_{\min }}{{\mathrm{x}}_{\max}\hbox{-} {\mathrm{x}}_{\min }} $$

This normalization procedure was previously found to result in more accurate classification results for stroke/control subjects classification than a regime based on centering and scaling [27].

A system identification method known as Fast Orthogonal Search (FOS) [28] was used to identify the most informative clinical and robotic metrics that contribute towards prediction of FIM scores at approximately 2 weeks and 3 months post stroke. FOS allows for identification of a non-linear approximation consisting of terms with the highest contribution towards prediction of a desired target value [28].

In this study, the measured FIM score at approximately 2 weeks and 3 months post stroke, denoted by S, was used as the target value in a FOS training algorithm to form a sum of *M* non-linear basis functions *p*_*m*_*(n)* with coefficients *a*_*m*_ and estimate error *e(n)* as follows

2 $$ S={\displaystyle {\sum}_{m=1}^M{a}_m\kern0.5em {\mathrm{p}}_m(n)+e(n)} $$

The FOS training algorithm searches through all “*N*” input candidate basis functions, where *N*> > *M* and iteratively picks those candidates which contribute the largest mean square error (MSE) error reduction between the model estimate and the actual data (*S*). This method is based on the theory of Gram-Schmidt orthogonal identification, whereby orthogonal basis functions are generated from the *p*_*m*_*(n)* and coefficients are found such that the MSE of the estimate is minimized [28]. The first function selected by any model using FOS is assigned a value of 1, with a coefficient term that accounts for the constant term in the model. Following this first iteration, all subsequent basis functions are chosen from the provided pool of candidates. Non-linearity was introduced into our predicted models by including *squared, cubic, sin, cosine,* and *logarithmic* functions of the robotic metrics. A detailed description of this method is presented in [28].

FIM-related predicted measures in this study were FIM-Motor and FIM-Total at entrance to the study approximately two weeks post-stroke (*FIM-Motor-2w* and *FIM-Total-2w*) and at approximately three months post-stroke (*FIM-Motor-3 m* and *FIM-Total-3 m*). If a score is predicted to be outside the valid range of FIM values (e.g. <18 and >126 for FIM-Total), the predicted value is adjusted to be the minimum or maximum valid score for scores below and above the valid range, respectively.

### Performance Measure

All models were evaluated by a 10-fold cross validation procedure using 90 % of the subjects’ data to train the model with the remaining 10 % for model testing. Overall model performance is the average of the performance of test data over the 10 folds.

To decide on the optimal number of parameters for model generalization a total of 15 models consisting of 1 to 15 basis functions were tested for each prediction. Consequently, the models that generalize best on unseen data were used as the optimal model. The addition of a higher number of basis functions results in a decrease in the models’ performance on unseen validation data due to model over-fitting. The optimal number of metrics for each estimated model is reported for each model.

Performance of predicted clinical measures is reported as the R value between the actual and predicted values. R is a value between 0 and 1 and can be interpreted as the correlation coefficient between actual and predicted values, indicating the “goodness of fit” for such values. Moreover, the value of *R*^2^ can be used to interpret the fraction of unexplained variance in data, since it compares the variance of the model’s errors with the total data variance.

### Datasets for model generation

We employed both robotic and clinical biomarkers to build predictive models. Robotic biomarkers included the *Reaching* task parameters recorded for the affected and unaffected arm, henceforth referred to as *Reach-affected* and *Reach-unaffected*. The set of reaching parameters describing the interlimb difference is referred to as *Reach-Interlimb*. A single set of data describes *Matching* task parameters, referred to as *Match*. Parameters associated with the *Object Hit* task are referred to as *Object Hit*. The last robotic dataset, *All Robotic Data*, contains all reaching, matching, and object hit parameters.

Clinical biomarkers are comprised of *FIM-2w* and a set including the Chedoke-McMaster Score, Purdue Pegboard Score, BIT Score and Modified Ashworth Score. This latter set included measures for both affected and unaffected arms, henceforth referred to as *Clinical-affected* and *Clinical-unaffected*. Another set containing both *Clinical-affected* and *Clinical-unaffected*, henceforth referred to as *Clinical-All,* was also used for model generation.

For the case of each FIM prediction, all parameters in the above mentioned sets of biomarkers (i.e. robotic or clinical) were used as the candidate basis functions for the FOS training algorithm as described above. The FOS training algorithm then picks the parameters within each dataset based on their contribution towards minimizing the prediction error (as measured by MSE) for prediction of each FIM score.

## Results

### Participant Pool

Demographics data on the participants’ age, gender, type of stroke, as well as the distribution of clinical scores is provided in Table 2. Clinical and robotic assessments were carried out on 85 patients with stroke (49 left- and 33 right-affected, 61 with ischemic and 24 with hemorrhagic stroke). The youngest participant was aged 25, and the oldest was 82 years old, and the average age was approximately 61 years. The range of clinical scores that are covered in this study are also presented in Table 2.

**Table 2:** Demographics and Clinical Data

Measure	Quantity
Number of Subjects	85
Male/Female	57/28
Affected Limb (Left-side/Right-side/Both)	49/33/3
Type of stroke (Ischemic/Hemorrhagic)	61/24
Age in years	(60.6, 12.5, 25, 82) ^a^
Days since stroke to first FIM	(8.1, 4.5, 1, 14) ^a^
Days since stroke to second FIM	(89.6, 8.9, 68, 125) ^a^
FIM-Motor-2w score	(66.5, 21.5, 13, 91) ^a^
FIM-Total-2w score	(96.3, 24.3, 37, 126) ^a^
FIM-Motor-3 m score	(85.9,10.1,27,91) ^a^
FIM -Motor-3 m score	(118.5,12.8,45,126) ^a^
Purdue Pegboard Score - Combined	(14.9, 6.0, 4, 28.5) ^a^
BIT score	(133.8, 19.1, 63, 146) ^a^
Modified Ashworth Score - Affected Arm	(70, 5, 4, 6, 0, 0)^b^
Modified Ashworth Score - Unaffected Arm	(83, 2, 0, 0, 0, 0)^b^
Chedoke McMaster Score - Affected Arm	(11, 9, 8, 3, 16, 8,30)^c^
Chedoke McMaster Score - Affected Hand	(14, 4, 5, 6, 16, 23, 16)^c^
Chedoke McMaster Score - Unaffected Arm	(0, 0, 0, 0, 4, 11, 70)^c^
Chedoke McMaster Score - Unaffected Hand	(0, 0, 0, 0, 3, 24, 58)^c^

^a^ Formatting represents (Average, Standard Deviation, Minimum, Maximum)

^b^ Formatting represents number of participants with Modified Ashworth Score of (0, 1, 1+, 2, 3, 4)

^c^ Formatting represents number of participants with Chedoke McMaster Score of (1, 2, 3, 4, 5, 6, 7)

### Estimates of FIM-2w Scores from Robotic and non-Robotic Data

Our first analysis focused on estimation of *FIM-2w* scores from robotic and non-robotic biomarkers. R values for estimation of *FIM-Motor-2w* and *FIM-Total-2w* scores are shown in Table 3. Our results show that the largest R values for prediction of *FIM-2w* scores are achieved using the Clinical-all dataset (0.829 and 0.817 for *FIM-Motor-2w* and *FIM-Total-2w*, respectively). R values for the robotic datasets also show high correlations of 0.742 and 0.774 for *FIM-Motor-2w* and *FIM-Total-2w*, respectively. R values for the Object Hit dataset are almost as large as for the *All Robotic Data*.

**Table 3:** R values for prediction of FIM-related scores at 2 weeks and 3 months post-stroke using clinical and robotic data (validation results)

		FIM-Motor-2w	FIM-Total-2w	FIM-Motor-3 m	FIM-Total-3 m
1	Reach-Affected	0.6804	0.7209	0.4205	0.4394
2	Reach-Unaffected	0.3985	0.4045	0.3524	0.3481
3	Reach-Interlimb	0.6324	0.6303	0.2471	0.2306
4	Match	0.4608	0.5219	0.3836	0.2825
5	Object-Hit	0.7237	0.7542	0.6221	0.5906
6	All Robotic Data	0.7417	0.7745	0.6936	0.6851
7	FIM-2w	--	--	0.6255	0.5987
10	Purdue-Combined	0.7230	0.7393	0.5031	0.5129
8	Chedoke-Affected	0.7310	0.7271	0.5399	0.5241
9	Chedoke-Unaffected	0.3083	0.3338	0.2266	0.2598
11	BIT score	0.3659	0.3894	0.3824	0.3808
12	Clinical-Affected	0.8279	0.8106	0.6013	0.6133
13	Clinical-Unaffected	0.5252	0.5310	0.3128	0.3563
14	Clinical-All	0.8287	0.8172	0.6258	0.6434

For dataset abbreviations (rows), see the Methods section

Table 4 displays statistical comparisons between R values obtained for some of the best datasets to predict *FIM-2w* scores. The difference in R values between *All Robotic Data* and *Clinical-All* is not significant (P > 0.05). Estimates of *FIM-Motor-2w* was better for the *Clinical-All* and the *Clinical-Affected* datasets than for the *Object Hit* dataset (*p* = 0.043 for *Clinical-All* and *p* = 0.045 for *Clinical-Affected*), but not for the *FIM-Total-2w*.

**Table 4:** P-values for comparison of R values between datasets

	Dataset 1	Dataset 2	FIM-Motor-2w	FIM-Total-2w	FIM-Motor-3 m	FIM-Total-3 m
1	Object-Hit	FIM-2w	--	--	0.484	0.468
2	Object-Hit	Clinical-All	**0.043**	0.145	0.484	0.291
3	All Robotic Data	FIM-2w	--	--	0.203	0.184
4	All Robotic Data	Clinical-All	0.074	0.215	0.203	0.333

p values < 0.05 is shown in bold

Table 5 and 6 shows the model parameters for predictions of *FIM-2w* scores using *All Robotic Data* (top) and *Clinical-All* (bottom). Our models generated by FOS generalize the best using 2 basis functions for both *FIM-Motor-2w* and *FIM-Total-2w* for the *Clinical-All* dataset. The models’ performance drops on unseen validation data when including higher number of functions due to model overfitting. Basis functions are reported in Table 5 and 6 by the order in which they are selected by FOS. Thus, the first chosen basis function is the one that contributes the most to the value of the estimated model. Repeat rates for each model parameter is generated by repetitively constructing optimal models using a randomly selected 90 % subset of the original data for 100 repeats and reporting how many times each metric is picked by FOS.

**Table 5:** Robotic Metrics for Prediction of FIM-related Scores and their Corresponding Repeat Rates out of 100 Repeats for Each Model Estimation

Top: predictions using robotic data													
	Number of model parameters	First Metric	Repeat rate (%)	Second Metric	Repeat rate (%)	Third Metric	Repeat rate (%)	Fourth Metric	Repeat rate (%)	Fifth Metric	Repeat rate (%)	Sixth Metric	Repeat rate (%)
FIM-Motor-2w	5	M3	81	OH11	26	M5	45	OH1	33	OH11	23	N/A	N/A
		RA5	12	OH12	16	OH1	24	M5	21	RA1	16		
		RA9	4	OH14	15	OH12	6	OH12	8	M9	10		
FIM-Total-2w	4	M3	95	M5	55	OH14	37	M9	28	N/A	N/A	N/A	N/A
		RA9	5	OH6	17	OH11	22	OH1	25				
				OH1	12	OH12	17	M5	19				
FIM-Motor-3 m	6	OH12	76	OH13	73	RA11	59	OH5	37	RA11	49	OH5	28
		RA4	11	RA4	18	OH13	16	RA11	12	OH5	16	RA4	18
		OH13	5	OH12	6	OH7	8	OH7	11	RA4	11	RA11	8
FIM-Total-3 m	5	OH12	75	OH13	78	RA11	63	RA7	34	M9	27	N/A	N/A
		OH11	10	RA11	9	OH5	7	OH7	12	OH5	18		
		OH9	9	OH12	8	RA7	5	M9	11	M6	11

**Table 6:** Robotic Metrics for Prediction of FIM-related Scores and their Corresponding Repeat Rates out of 100 Repeats for Each Model Estimation

Bottom: predictions using clinical data							
	Number of model parameters	First Metric	Repeat rate (%)	Second Metric	Repeat rate (%)	Third Metric	Repeat rate (%)
FIM-Motor-2w	2	CM-A	100	PP	98	N/A	N/A
				CM-U	2		
FIM-Total-2w	2	CM-A	97	PP	96	N/A	N/A
		PP	3	CM-A	3		
				CM-U	1		
FIM-Motor-3 m	3	PP	100	CM-A	85	PP	75
				CM-U	15	CM-A	16
						CM-U	10
FIM-Total-3 m	3	PP	100	CM-A	89	CM-A	43
				PP	7	CM-U	37
				CM-U	4	PP	20

*PP* Purdue Pegboard Score- Combined, *CM-A* Chedoke-McMaster Score-Affected Arm, CM-U Chedoke-McMaster Score-Unaffected Arm

For abbreviations of robotic parameters, see Table 1.

For *FIM-Motor-2w* and *FIM-Total-2w*, the first dominant metric contributing to the model estimation is the Chedoke-McMaster Score-Affected, and the second dominant metric is associated with the Purdue Pegboard Score.

For predictions of FIM scores using robotic data, the best generated models generalize best using four and five parameters for *FIM-Total-2w* and *FIM-Motor-2w*, respectively. The first dominant metric contributing to the estimation of both *FIM-Total-2w* and *FIM-Motor-2w* is associated with the arm-position *Matching* task *(M3: Variability XY).* The second and third dominant metrics are also parameters associated with arm-position *Matching* task *(M5: Contraction/Expansion XY)* and two metrics associated with the *Object-Hit* task *(OH11: Hand speed bias, OH14: Movement Area Bias).*

### Estimates of FIM-3 m Scores from Robotic and non-Robotic Data

We next examined the estimation of *FIM-3 m* scores from robotic and non-robotic biomarkers. The best performance in terms of R values for prediction of *FIM-3 m* scores are achieved using the *All Robotic* datasets (0.694 and 0.685 for *FIM-Motor-3 m* and *FIM-Total-3 m*, respectively). This is followed by the R values obtained using the *Clinical-All* dataset (0.626 and 0.643 for *FIM-Motor-3 m* and *FIM-Total-3 m*, respectively). Also predictions of *FIM-Motor-3 m* and *FIM-Total-3 m* using the respective *FIM-2w* scores had R values of 0.626 and 0.599, respectively. Table 4 highlights that there are no statistical differences between predictions of FIM-3 m scores generated from the *Clinical-All*, *All Robotic Data* and the *FIM-2w* datasets.

Table 5 and 6 displays the key biomarkers that contribute to each model estimation. For *FIM-Motor-3 m* and *FIM-Total-3 m*, the dominant first and second metrics are associated with the *Object Hit* task for both model estimations, while the third dominant parameter is associated with the *Reaching* task (affected side data). This is consistent with our results in Table 3, where *Object Hit* parameters resulted in models that generalized better for prediction of FIM scores at both 2 weeks and three months post-stroke. The dominant *Object Hit* parameters include *Movement Area-Affected Arm (OH12)* and *Movement Area-Unaffected Arm (OH13)* for both *FIM-Motor-3 m* and *FIM-Total-3 m* predictions. *Reaching* task parameters used for both predictions include *First Movement Direction error (RA4), First Movement Maximum Speed Ratio (RA11)*, and *Path Length Ratio (RA7).*

Models that used clinical data for estimation of *FIM-Motor-3 m* and *FIM-Total-3 m* scores generalize with three parameters for both scores. Similar to predictions of scores at 2 weeks post stroke, *FIM-3 m* estimations are best predicted using a combination of Purdue Pegboard and Chedoke-McMaster assessment scores. However, for *FIM-3 m* predictions the first dominant metric is associated with the Purdue Pegboard test for both *FIM-Motor-3 m* and *FIM-Total-3 m*. The second dominant metric is associated with the Chedoke-MacMaster score, affected side. These results are consistent with our finding presented in Table 7, where both Purdue Pegboard and Chedoke MacMaster-affected side show moderate correlation values for prediction of *FIM-3 m* scores.

**Table 7:** R Values on Validation Data for Robot-Based Models to Predict Other Clinical Scores and the Robotic Metrics Associated with the Model Based on All Robotic Data

	Reaching	Matching	Object Hit	All Robotic Data	First Metric	Repeat rate (%)	Second Metric	Repeat rate (%)	Third Metric	Repeat rate (%)
Purdue-Combined	0.778	0.567	0.698	0.791	RA12	61 %	OH1	48 %	M9	31 %
					OH1	28 %	RA2	24 %	OH8	21 %
					RA5	10 %	M3	17 %	RA3	12 %
CMS-Affected	0.775	0.394	0.714	0.810	RA5	63 %	RA3	46 %	OH13	39 %
					R12	21 %	OH8	31 %	M1	28 %
					RA3	11 %	RA2	13 %	RA2	19 %
CMS-Unaffected	0.096(NS)	0.006(NS)	0.043(NS)	0.097(NS)	OH1	18 %	OH2	21 %	N/A	N/A
					M8	16 %	M4	13 %		
					M4	12 %	RA5	10 %		
MAS-Affected	0.211	0.052(NS)	0.038(NS)	0.235	RA1	45 %	OH8	30 %	N/A	N/A
					OH8	21 %	RA9	22 %		
					OH1	13 %	M8	10 %		
MAS-Unaffected	0.193(NS)	0.063(NS)	0.233	0.266	OH14	32 %	RA12	27 %	N/A	N/A
					OH7	24 %	M1	19 %		
					RA12	11 %	RA5	11 %		
BIT Score	0.510	0.488	0.415	0.678	M2	48 %	RA6	38 %	OH8	31 %
					RA11	30 %	RA3	26 %	OH1	25 %
					RA3	14 %	M2	20 %	M1	17 %

*Matching* Arm position matching task, *Reaching* visually-guided reaching task, affected, unaffected and interlimb parameters, *Object Hit* Ball drop object hit task, *CMS* Chedoke-MacMaster Score, *MAS* Modified Ashworth Score, *NS* Not significant for *P* < 0.005. For abbreviations of robotic metrics see Table 1

### Estimating Clinical Scores from Robotic Data

Our next analysis examines the relationship between robotic and clinical measures of upper limb function. Table 7 shows validation results (data not used to develop the models) using robot-based tasks to estimate the traditional clinical scores using *Reaching*, *Matching*, *Object Hit*, and *All Robotic* data. R values for prediction of Purdue-Combined and CMS-Affected using *Reaching* task parameters show high correlations (0.778 and 0.775). Predictions using *All Robotic Data* are slightly improved by inclusion of *Object Hit* and *Matching* data (0.791 and 0.810). Also the predictions of BIT score using robotic data shows moderate correlations with each set of robotic task data and a relatively high R value of 0.678 when using *All Robotic Data*. All R values except for the Chedoke-McMaster Score-Unaffected were significant at *P* < 0.005. The correlation values for the Modified Ashworth Score are low (R = 0.235 and 0.266 for affected and unaffected arms, respectively). This is in line with the findings in [29] for the Modified Ashworth score, where they report R = 0.08-0.17 for validation data for predictions made using robot-based kinematics.

Table 7 also shows the key robotic biomarkers for prediction of these clinical scores. The dominant first and second robotic metrics for prediction of Purdue-Combined and CMS-Affected are both associated with the *Reaching (RA12* and *RA5)* and *Object Hit (OH1* and *OH8)* task, while there is some small contribution from the *Matching* task as the third metric. For prediction of BIT score, the major contributions are from the *Matching* (*M2*) and *Reaching* task (*RA11* and *RA6*), with smaller contributions from the *Object Hit* tasks as the third metric.

## Discussion

The purpose of the present study was to identify the biomarkers of sensory and motor function of the arms at approximately 2 weeks post-stroke to estimate present and future FIM scores. Our results highlight that robotic and non-robotic assessments were not statistically different when predicting *FIM-2w* and *FIM-3 m* scores. There was a general trend towards better estimates for clinical biomarkers to predict *FIM-2w* scores and robotic biomarkers to predict *FIM-3 m* scores. Importantly, robot-based biomarkers of proprioceptive function were influential for predicting FIM scores at 2 weeks, whereas biomarkers related to rapid bimanual motor function were influential for predicting FIM scores at 3 months.

Robot-based measures predicted Purdue Pegboard and Chedoke-McMaster Scores with high R values of ~0.8 for the affected limb within approximately 2 weeks post-stroke. Our predicted R value for the Chedoke-McMaster score is statistically higher than the results obtained in [29] for robot-based estimates of the Fugl-Meyer Assessment and Motor Power (0.427 and 0.449 respectively, on validation data). The fact that the prediction of these two clinical scores is attributed to robotic parameters associated with the *Reaching* and *Object Hit* tasks is in line with the observation that these two clinical tests are predominantly dependant on motor skills. Our improved estimates likely reflect two distinct factors. First, our mathematical models using FOS can consider non-linear relationships rather than simple linear regressions. Secondly, our range of robotic tasks covered a broader range of assessments of brain deficits including bimanual rapid visuomotor and proprioceptive tasks. While correlations were generated largely from measures of visual-guided reaching performance, there was some contribution from the limb matching and object hit tasks.

R values associated with prediction of BIT scores are also relatively high (0.678 using all sets of robotic data). Previous studies have reported significant correlations between BIT and some robot-based metrics associated with motor function [30] (correlations as high as 0.50) and proprioceptive deficits [31] (correlations as high as -0.55) following stroke. In the present study we directly attempted to develop models to predict measures of spatial neglect using robot-based evaluation metrics for subjects with stroke. The correlation associated with BIT was attributed to measures of limb position matching and reaching performance with a smaller amount of contribution from the object hit task. This is in line with the inherent characteristics of reaching task, which quantifies reaching performance across targets at 8 different spatial locations and limb position matching task, which assesses sense of position across 9 different spatial targets.

Predictions of Modified Ashworth Scores resulted in poor prediction performance for both affected and unaffected limbs (0.235 and 0.266, respectively). These results conform to the findings of [29] (0.171 on validation data), although our R values are found to be statistically significant for *P* < 0.005, which may be attributed to our non-linear modeling approach.

The ability of non-robotic assessment measures to predict *FIM-2w* scores better than robot-based measures may be related to the impact of weakness on subject performance. Weakness is commonly observed post-stroke, and when severe, makes it impossible to perform any motor activity due to gravitational forces impeding goal-direct movements of the limb. This would directly impact the ability to perform daily activities as measured by FIM, as well as performance in the Purdue pegboard test and the Chedoke-McMaster test. In contrast, the robotic device provided weight support of the arms. Thus, a subject unable to lift their arms due to weakness may still be able to perform reaching and object hit movements to a certain degree.

It is interesting to note that the dominant robot-based biomarker to predict *FIM-2w* scores was related to proprioceptive function from the arm position matching task. Our previous study using this task identified high correlation between FIM scores and matching task parameters [25]. This suggests that proprioceptive function is important to plan and control many activities of daily living, such as grooming, eating and bathing. Moreover, proprioceptive deficits in the arm and leg are correlated [32], and thus, may indirectly predict difficulties in performing lower limb activities such as locomotion. Also, it has been noted previously that proprioceptive deficits do not correlate with motor skills such as reaching post stroke [9]. This explains the presence of additional non-matching task parameters in models for predicting *FIM-2w* scores.

Over the course of rehabilitation between first and second FIM measurements, the prediction of *FIM-3 m* scores using robot-based measures was slightly, although not statistically, better than clinical scores and FIM scores measured at 2 weeks. The dominant parameters attributed to the prediction of *FIM-3 m* are associated with the object hit task*.* The selected parameters in the robot-based models provide some insight as to the relative success of the robot-assessment to predict future abilities to perform daily activities. First, Movement Area by both affected and unaffected hands from the object hit task were the most commonly selected parameters when developing models to predict future FIM scores. This is consistent with our understanding of the ability to perform daily activities, as the wider span of hand movements can prove to amount to a higher level of independence during activities of daily living. In general, dominance of object hit task parameters for prediction of future FIM scores highlights the fact that weakness in moving would amount to subjects not being able to quickly move arms for success on the object hit task. Moreover, the majority of parameters on the object task have inter-rater reliability scores above the statistically accepted norm of 0.8 [14].

Second, the relative success to predict future FIM scores may be related to the fact that robot-based models commonly selected parameters that characterize differences in limb movement, measures that compared object hit performance between the affected and unaffected limb. This includes parameters such as Miss bias, Hand speed bias and Hand selection overlap. These interlimb measures neutralize differences in each subject’s motor strategy, making it easier to capture differences in motor capabilities between the limbs.

Third, the fact that the robot provided weight support for the limb may have reduced their ability to predict *FIM-2w* scores, but paradoxically may have improved its ability to predict *FIM 3 m* scores. Many subjects with stroke and motor deficits will improve strength with time. By alleviating weight support, the robot-based tasks assess other aspects of sensorimotor function beyond strength, allowing us to uncover other important impairments that can impact long term abilities to perform daily activities.

A recent study similar in nature to the present study was performed by Krebs, et al. [33]. They used a neural network technique to construct mathematical models for prediction of a set of clinical scores including Fugl-Meyer, National Institute of Health Stroke Scale, and Motor Power based on robotic measurements. This study was performed for a group of “completers” (who had complete data for days 7 and 90 after stroke) and “non-completers” (who had some missing data on day 7 or 90). The main difference between that study and the present one is that the robotic data used for model generation for that study are collected at the same time as robotic evaluation. In contrast, our study uses data collected at day 14 to make predictive models on FIM measurements approximately 3 months post stroke. Reported R^2^ values in that study range from 0.45–0.6 for the “non-completer” group and 0.58–0.75 for the “completer” group. Thus, our estimates of future FIM scores are relatively good given the challenge of predicting FIM scores several months in the future and following rehabilitation therapy.

One of the limitations of our present study is the absence of behavioral tasks for assessment of cognitive brain function. FIM-Total is comprised of 5 items for assessment of cognition including memory, problem solving, and concentration. Robot-based predictions of *FIM-Total-2w* tended to be better for than for *FIM-Motor-2w*, suggesting cognitive impairments likely impact performance in these tasks. Importantly, one of the advantages provided by robotic assessment is the potential to study behavioral tasks to quantify impairments in a broad range of sensory, motor and cognitive functions. We have recently developed several other tasks to assess sense of limb motion [10], ability to use sensory feedback for postural control [34] and bimanual motor function [35]. As well, we have developed more cognitively demanding tasks on the robotic system. An extension of the object hit task used in the present study is an object hit and avoid task, which requires additional cognitive processing to attend to the appropriate targets and inhibitory control to avoid distractors present in the workspace. Moreover, the pen-and-paper Trail Making test [36] that examines task switching has also been implemented on the robotic platform. When data on these tasks are added to the analysis, we expect that we will be able to make improved predictions on the ability of subjects to perform daily activities, as measured by the FIM, both at the present and in future time points.

One of the advantages of using robotic technologies for prediction of FIM scores lies in the ability to discover the parameters that are most selected in the models to improve the predictions of FIM. This has the potential to ultimately lead to a better understanding of the underlying changes through the rehabilitation process that may, in turn, lead to improved patient care.

Given the limited amount of available data and related studies in the use of robotic technologies for post-stroke neurorehabilitation, our collective analysis suggests that robotic technologies may enable early decision support in clinical assessment, reduce the amount of time required for assessment, and offer a reliable tool for monitoring of longitudinal changes for stroke survivors in conjunction with the current clinical scales. With addition of extra data for assessment of other areas of brain dysfunction, we expect to come up with improved decisions for patient care. The present study serves as a proof of principle, with the addition of other forms of data providing extra benefits.

## Conclusions

The present study used biomarkers generated from a set of three behavioral robotic tasks to estimate measures of daily activities pre- and post-rehabilitation. Findings of the study highlight that robot-based metrics provide an accurate estimate of future FIM scores, in line with estimates provided by clinical scores at approximately 2 weeks post-stroke. While the findings of this study provide a proof of principle for use of robotic tools in clinical decision support, addition of other behavioral tasks on the robotic settings is expected to provide more accurate predictions in the future.

## Abbreviations

FOS: fast orthogonal search
FIM: functional independence measure
FIM-2w: functional independence measure assessed 2 weeks post-stroke
FIM-3 m: functional independence measure assessed 3 months post-stroke
CMS: chedoke mcmaster score
MAS: modified ashworth score
BIT: behavioral inattention test
KINARM: kinseological instrument for normal and altered reaching movement
